# Factors associated with direct support professionals' behaviour in the physical activity support provided to people with intellectual disabilities

**DOI:** 10.1111/jir.12616

**Published:** 2019-04-03

**Authors:** L. W. M. Bossink, A. A. J. van der Putten, M. C. S. Paap, C. Vlaskamp

**Affiliations:** ^1^ Department of Special Needs, Education, and Youth Care University of Groningen Groningen The Netherlands

**Keywords:** direct support professionals, intellectual disability, physical activity promotion, professional behaviour, professional support

## Abstract

**Background:**

Direct support professionals play an important role in facilitating physical activity support for people with intellectual disabilities (ID). This study examined how the characteristics of people with ID and the characteristics of direct support professionals are related to the professionals' behaviour when supporting people with ID in physical activity.

**Methods:**

A cross‐sectional approach was used. Direct support professionals (*n* = 217) who support people with ID completed a self‐report questionnaire, which aimed to measure the components that produced behaviour when providing physical activity support for people with ID. Associations with the characteristics of people with ID and the characteristics of the professionals were analysed using multivariate linear regression models.

**Results:**

The results demonstrate that the professionals' characteristics – such as age, workplace and training – were related to the variance in the components that theoretically produced the direct support professionals' behaviour. The characteristics of the people with ID did not contribute to the variance in the direct support professionals' behaviour.

**Conclusions:**

The findings suggest that professional characteristics are the dominant reasons for the differences observed in the capability, opportunity and motivation of direct support professionals to provide physical activity support. This study also underscores the need for integrated training programmes to help direct support professionals promote physical activity in people with ID.

## Background

Physical activity provides various benefits to people with intellectual disabilities (ID) (Bartlo & Klein 2011; Houwen *et al*. 2014). Bartlo and Klein (2011) summarised the evidence for adults with ID in a systematic review and found that physical activity positively affects balance, muscle strength and quality of life in adults with ID. Houwen *et al*. (2014) also reviewed the evidence for adults with severe or profound ID and found that all the included studies of the effectiveness of participating in movement‐oriented activities reported beneficial effects in the motor domain. Several studies, however, suggested that physical inactivity is common in people with ID (Dairo *et al*. 2016; Stancliffe & Anderson 2017). People with severe or profound ID appear to be at particularly high risk of being physically inactive and at even greater risk when these disabilities are present along with severe motor disabilities (Dairo *et al*. 2016; Stancliffe & Anderson 2017; Van der Putten *et al*. 2017). In addition, it is known that the level of engagement in physical activity in people with ID decreases as an individual ages (Robertson *et al*. 2000; Dairo *et al*. 2016; Stancliffe & Anderson 2017; Van der Putten *et al*. 2017) and develops more health problems (Robertson *et al*. 2000; Stancliffe & Anderson 2017).

The results of a systematic review to identify the barriers to and facilitators of physical activity in people with ID reveal a broad range of barriers and facilitators (Bossink *et al*. 2017). Numerous environmental barriers were found for people with ID, many of which were related to the professional support provided (e.g. lack of staff expertise and interest and resistance to change established work routines). Moreover, professional support has been shown to predict physical activity levels in adults with ID (Peterson *et al*. 2008). Addressing physical inactivity in people with ID could therefore include targeting the professional behaviour of those who are likely to be tasked with supporting people with ID to engage in physical activity.

So far, only limited data are available that focus on the professional behaviour of direct support professionals in the physical activity support they provide to people with ID. We conducted an initial qualitative study with direct support professionals to explore what influence their behaviour has in supporting people with ID to engage in physical activity (Michie *et al*. 2005). The results suggested 30 influential factors that facilitate or impede the support of physical activity by direct support professionals (Bossink *et al. under review*). In this study, we used the Theoretical Domains Framework because it provides a methodology for understanding a specific behaviour theoretically and can be condensed into a ‘behaviour system’ involving three core components: capability, opportunity and motivation (the ‘COM‐B system’) (Michie *et al*. 2011; Cane *et al*. 2012). The study showed that direct support professionals experience factors that impact on their capability to support people with ID to engage in physical activity, the opportunities afforded them and subsequently their motivation (Bossink *et al. under review*). This qualitative study suggested that specific characteristics of the people with ID (i.e. intellectual and physical disabilities, their physical and mental health, age and their preferences or motivation) greatly affect the direct support professionals' capability, motivation and the opportunities afforded them (Bossink *et al. under review*).

Although the aforementioned results demonstrate an important overview of potential factors that influence direct support professionals' behaviour, they do not provide us with any quantified insight into these essential components (capability, opportunity and motivation), which produce behaviour and their relationship with the characteristics of people with ID. Furthermore, because experiences differed in our qualitative study, the extent to which the direct support professionals' own characteristics relate to their capability, opportunity or motivation to provide physical activity support can be questioned. Previous studies have suggested some professional characteristics that are thought to contribute to the degree to which direct support professionals actively engage an individual with ID in daily activities. The professional characteristics associated with supporting people with ID in healthy daily activities in past studies include professional qualification (Robertson *et al*. 2000; Mansell *et al*. 2008; Qian *et al*. 2015), the receipt of additional training or support (Jones *et al*. 1999; Jones *et al*. 2001; Mansell *et al*. 2003; Mansell *et al*. 2008; O'Leary *et al*. 2017), the work environment (Qian *et al*. 2015) and the years of experience as a direct support professional (Felce *et al*. 2002). However, these characteristics have not been studied in relation to physical activity support.

Against this background, this study's aim is to examine the extent to which the characteristics of people with ID and their carers' professional characteristics contribute to the variance observed in the capability, opportunity and motivation of direct support professionals to provide physical activity support. Understanding the factors associated with direct support professionals' behaviour can have important implications for planning more tailored policies to reduce physical inactivity in people with ID.

## Methods

### Study design and participant selection

A cross‐sectional approach was used. The participants were recruited by convenience sampling using the following inclusion criteria: (1) being a professional supporting a group of people with ID in a living unit and/or activity centre and (2) being directly in contact with people with ID for most of the time at work. No reward or incentive was offered for participation. The sample used in this study shows overlap with that used in a previous study (Bossink *et al. under review*). However, the whole sample (*N* = 247) was used in the previous study, whereas participants were excluded from the current study if they had completed less than half of the demographic questionnaire. This yielded a sample of 217 participants for this study. The participants providing direct and daily support to people with ID came from 26 different healthcare organisations. The participants aged between 22 and 65 years [Mean = 42.4; standard deviation (SD) = 11.6]. A total of 84% were female. The level of ID of the people with ID supported by the participants varied from mild (20%), moderate (30%), severe (24%) to profound (21%). Table 1 details the characteristics of the participants and of the people with ID.

**Table 1:** Sample characteristics

Characteristic^a^		
Participant		
Age (*n* = 213)	Years, Mean (SD), range	42.4 (11.6), 22–65
Gender (*n* = 215)	Female, *n* (%)	181 (84)
Profession‡ (*n* = 216)	Direct support professional, *n* (%)	93 (43)
	Senior direct support professional, *n* (%)	123 (57)
Duration of contact (*n* = 216)	Hours per week, Mean (SD), range	26.1 (6.4), 6–40
Work experience (*n* = 215)	Years, Mean (SD), range	16.6 (10.2), 1–46
Education level (*n* = 216)	Vocational education, *n* (%)	133 (62)
	Higher professional education or master's, *n* (%)	83 (38)
Training in physical activity support (*n* = 213)	Yes, *n* (%)	57 (27)
Workplace (*n* = 209)	Living unit, *n* (%)	147 (70)
	Activity centre, *n* (%)	41 (20)
	Both, *n* (%)	21 (10)
Internal support for physical activity (*n* = 213)	Yes, *n* (%)	124 (58)
Physical activity plan for all people with ID§ (*n* = 191)	Yes, *n* (%)	63 (33)
	Mixed, *n* (%)	49 (26)
People with ID the participants support		
Level of intellectual disability (*n* = 217)	Mild, *n* (%)	44 (20)
	Moderate, *n* (%)	65 (30)
	Severe, *n* (%)	52 (24)
	Profound, *n* (%)	47 (21)
	All applicable, *n* (%)	9 (4)
Age group§ (*n* = 217)	≤18 years, *n* (%)	26 (12)
	19–37 years, *n* (%)	42 (19)
	38–57 years, *n* (%)	78 (36)
	≥58 years, *n* (%)	32 (15)
	Various, *n* (%)	39 (18)
Motor disability (*n* = 216)	Yes, *n* (%)	154 (71)
Visual impairments (*n* = 216)	Yes, *n* (%)	108 (50)
Auditory impairments (*n* = 214)	Yes, *n* (%)	63 (30)
Health problems (*n* = 217)	Yes, *n* (%)	156 (72)
Mental health problems/challenging behaviour (*n* = 217)	Yes, *n* (%)	187 (86)

a Not all the categories have the same total *n*, as a different number of responses were missing for each question.

‡ Senior direct support professionals have additional tasks such as coordinating the planning of multidisciplinary meetings, parental contact and partial responsibility for the content of individual support plans, etc.

§ We assigned a ‘various’ score to participants who indicated that more than two age groups were applicable.

### Data collection

The data were collected online using a 41‐item self‐report questionnaire that aimed to measure direct support professionals' behavioural determinants in physical activity support for people with ID. The questionnaire provides insight into three theoretical sources of behaviour (Michie *et al*. 2011): direct support professionals' capability, the opportunities afforded to them to provide support and their motivation to perform physical activity support. Capability was covered with a 7‐item scale, opportunity with a 16‐item scale and motivation with an 18‐item scale. Direct support professionals were asked to indicate their degree of agreement with the items based on a 5‐point Likert scale (from disagree to agree). Adequate psychometric evidence of the instrument was obtained in a previous study (Bossink *et al. under review*), with good construct validity and satisfactory marginal reliability coefficients (ranging from 0.84 to 0.87).

A self‐report demographic questionnaire was used to collect data about the people with ID and the characteristics of the professionals. *Qualtrics research software* was used for the online data collection.

### Independent variables

Potential explanatory variables were selected based a priori on previous research, clinical relevance and experience. The potential explanatory variables *related to the people with ID* were level of ID (mild, moderate, severe or profound), age group (≤18, 19–37, 38–57 and ≥58 years) and the presence or absence of additional motor, visual and auditory impairments, health problems and mental health problems or challenging behaviour. We excluded cases where the participants indicated that multiple categories were possible for the age group and ID level scores.

The potential explanatory variables *related to the professionals' characteristics* were gender, age (in years), educational level (vocational education vs. higher professional education or master's degree), profession (direct support professional vs. senior direct support professional), duration of contact (in hours per week), work experience as a direct support professional of people with ID (in years) and workplace (living unit vs. activity centre vs. both). Receipt of additional training in physical activity support or internal support provided by a physical therapist or a movement therapist was also included as a potential explanatory variable. Finally, the presence or absence of physical activity plans for the people with ID was included as an explanatory variable. The scores on for the variable ‘presence or absence of physical activity plans for people with ID’ were calculated using three continuous variables: the group size, the number of people with a physical activity plan and the number of people without a physical activity plan. We assigned a ‘yes’ score to participants who indicated that at least 70% of the people they supported had physical activity plans, a ‘no’ score to participants indicating that at least 70% of the people they supported did not have such plans and a ‘mixed’ score to the remainder of the participants.

### Statistical analysis

The participants' latent trait scores on the three subscales were estimated with IRT models using the parameters found in the study by Bossink *et al*. (*under review*). A graded response model, which can be regarded as a polytomous extension of the two‐parameter logistic model, was used to relate the item responses to latent trait values (Samejima, 1969). Three latent trait estimates were obtained for each person: scores on capability, opportunities and motivation. A latent score of 0 represents the mean of the IRT model sample. The variance (and thus the SD) of the latent scores was set to 1. Latent trait scores (theta scores) can thus be interpreted similarly to *z*‐scores.

Point estimates and plausible values were calculated for each latent trait. Using plausible values rather than point estimates (‘best estimates’) in subsequent analyses permits consideration of the measurement error associated with an estimated latent score for each person. This approach results in a more accurate estimate of standard errors for regression coefficients (see, e.g. Levy & Mislevy 2016; Fischer *et al*. 2018). Ten plausible values were sampled for each participant from the participant's posterior distribution.

Descriptive statistics were used to describe the sample characteristics. The mean (SD) scores for capability, opportunity and motivation were computed for the various subgroups of the categorical explanatory variables. A series of univariate linear regressions were fitted to identify significant explanatory variables. The multivariate linear regression models were fitted separately for the capability, opportunity and motivation scales. A stepwise approach was used for selecting the explanatory variables for the final regression models. First, a reference model was defined, composed of a set of explanatory variables related to the characteristics of people with ID. We proceeded by manually and sequentially removing the explanatory variables using a backwards elimination method (from least to most significant, with the removal criterion a *P* value > 0.10). Second, a reference model was defined comprising a set of explanatory variables related to the professionals' characteristics. We again proceeded by manually and sequentially removing the explanatory variables using a backward elimination method (from least to most significant, with the removal criterion a *P* value > 0.10). Third, the variables retained in the first two steps were entered together. Finally, backwards elimination was manually and subsequently applied again, until the final regression models only included significant explanatory variables (*P* value ≤ 0.05).

At each step, the assumptions of normality, homoscedasticity and the independence of the residuals were checked using the point estimates of the latent trait scores. We also checked for multicollinearity. Plausible values were used to evaluate whether the uncertainty associated with the estimated participants' latent trait scores used in this study had an impact on the results.

All statistical analyses were coded and performed using the open‐source software environment R version 3.4.3 (R Development Core Team, 2017). The graded response model was estimated using a full‐information maximum likelihood approach using the R mirt package version 1.27.1 (Chalmers *et al*., 2018); this package was also used to obtain plausible values. The R Zelig package version 5.1.6 was used to estimate the multivariate linear regression models using plausible values (Choirat *et al*. 2018), and the R car package version 3.0‐0 was used to check the model assumptions (Fox *et al*. 2018).

## Results

### Associations between the characteristics of people with intellectual disabilities and the behaviour components

Table 2 presents the mean latent trait scores (SD) for various categorical explanatory variable levels related to the characteristics of people with ID. Higher latent trait scores reflect greater capability, opportunities and motivation to engage in physical activity support. Table 2 also shows the corresponding results of the univariate linear regression analyses for the three subscales.

**Table 2:** Mean latent trait scores (SD) for capability, opportunity and motivation for the different levels of characteristics of people with ID and the results of the univariate linear regression analyses

Explanatory variables	Capability			Opportunity			Motivation		
		Univariate			Univariate			Univariate	
	Mean (SD)	Coefficient (SE)	*P*	Mean (SD)	Coefficient (SE)	*P*	Mean (SD)	Coefficient (SE)	*P*
Level of intellectual disability									
Mild	−0.25 (1.02)	Reference		−0.27 (0.94)	Reference		−0.21 (0.96)	Reference	
Moderate	−0.07 (0.83)	0.18 (0.171)	0.297	−0.10 (0.86)	0.17 (0.175)	0.339	−0.01 (0.88)	0.20 (0.178)	0.260
Severe	0.07 (0.81)	0.32 (0.180)	0.076*	0.25 (0.85)	0.51 (0.183)	0.006*	0.27 (0.99)	0.48 (0.187)	0.011*
Profound	0.09 (0.88)	0.34 (0.184)	0.065*	0.12 (0.94)	0.38 (0.188)	0.042*	0.06 (0.82)	0.27 (0.191)	0.156
Age group (years)									
≤18	−0.42 (0.82)	Reference		−0.49 (0.80)	Reference		−0.39 (0.80)	Reference	
19–37	0.13 (0.96)	0.55 (0.219)	0.012*	0.07 (0.89)	0.56 (0.228)	0.014*	0.19 (1.07)	0.58 (0.237)	0.016*
38–57	−0.02 (0.85)	0.40 (0.199)	0.049*	0.05 (0.96)	0.54 (0.207)	0.010*	0.05 (0.95)	0.43 (0.215)	0.045*
≥58	0.05 (0.87)	0.47 (0.231)	0.044*	−0.20 (0.91)	0.30 (0.241)	0.222	0.21 (0.89)	0.60 (0.251)	0.019*
Motor disability									
No	−0.17 (0.89)	Reference		−0.26 (0.86)	Reference		−0.09 (0.91)	Reference	
Yes	0.07 (0.91)	0.24 (0.136)	0.083*	0.14 (0.93)	0.40 (0.137)	0.004*	0.10 (0.94)	0.19 (0.141)	0.172
Visual impairments									
No	−0.10 (0.85)	Reference		−0.11 (0.88)	Reference		−0.07 (0.93)	Reference	
Yes	0.11 (0.95)	0.21 (0.123)	0.093*	0.16 (0.95)	0.27 (0.125)	0.032*	0.16 (0.93)	0.22 (0.127)	0.080*
Auditory impairments									
No	−0.01 (0.90)	Reference		0.00 (0.88)	Reference		0.01 (0.93)	Reference	
Yes	0.03 (0.95)	0.03 (0.137)	0.803	0.11 (1.05)	0.11 (0.139)	0.443	0.11 (0.96)	0.10 (0.141)	0.496
Health problems									
No	−0.07 (0.94)	Reference		−0.01 (0.90)	Reference		0.01 (0.91)	Reference	
Yes	0.04 (0.91)	0.11 (0.138)	0.438	0.05 (0.94)	0.06 (141)	0.688	0.07 (0.95)	0.06 (0.142)	0.663
Challenging behaviour									
No	0.29 (1.08)	Reference		0.11 (0.90)	Reference		0.15 (1.10)	Reference	
Yes	−0.03 (0.88)	−0.32 (0.179)	0.076*	0.02 (0.94)	−0.09 (0.183)	0.622	0.04 (0.91)	−0.11 (0.185)	0.554

* *P* ≤ 0.10.

The results show that the capability scores were higher on average in the participant groups supporting people with severe and profound ID, respectively, compared with the participant group supporting people with mild ID. The capability scores were on average lower in the participants supporting people with challenging behaviour, compared with the participants supporting people without challenging behaviour. In addition, significant associations (≤0.10) were found between capability and the groups characterised by age, motor disability and visual impairments.

Univariate analyses of the opportunity scores showed significant associations with ID level, age group and additional motor and visual impairments. The opportunity score was higher on average in the participant group supporting people with severe and profound ID, in the participant group supporting people within the 19–37 and 38–57 age groups, in the participant group supporting people with motor disabilities and in the participant group supporting people with visual impairments.

For motivation, significant associations were found with ID level, age group and visual impairments. The motivation score was on average higher in the participant groups supporting people within the 19–37, 38–57 and older than 57 age groups. In addition, the results showed that the participant group supporting people with severe ID scored higher on average for motivation compared with the group supporting people with mild ID, and a higher mean motivation score was found for the participants supporting people with visual impairments.

### Associations between the professional characteristics and the behaviour components

Table 3 presents the results of the univariate linear regression analyses for the associations between the professional characteristics and the three subscales. It also presents the corresponding mean latent trait score (SD) for the three subscales by the subgroups for each level of the categorical explanatory variables.

**Table 3:** Mean latent trait scores (SD) for capability, opportunity and motivation for the different levels of the professionals' characteristics, and the results of the univariate linear regression analyses.

Explanatory variables	Capability			Opportunity			Motivation		
		Univariate			Univariate			Univariate	
	Mean (SD)	Coefficient (SE)	*P*	Mean (SD)	Coefficient (SE)	*P*	Mean (SD)	Coefficient (SE)	*P*
Gender									
Male	0.00 (0.95)	Reference		0.19 (0.80)	Reference		0.03 (0.94)	Reference	
Female	0.01 (0.91)	0.01 (0.171)	0.949	0.00 (0.95)	−0.19 (0.174)	0.273	0.04 (0.94)	0.02 (0.175)	0.928
Profession									
DSP	−0.01 (0.93)	Reference		0.00 (0.91)	Reference		−0.02 (1.00)	Reference	
Senior DSP	0.02 (0.90)	0.04 (0.126)	0.779	0.06 (0.95)	0.06 (0.128)	0.636	0.09 (0.88)	0.11 (0.129)	0.392
Educational level									
Vocational education	0.06 (0.84)	Reference		0.15 (0.94)	Reference		0.07 (0.93)	Reference	
≥Higher professional education	−0.08 (1.02)	−0.15 (0.128)	0.248	−0.15 (0.89)	−0.30 (0.129)	0.021*	0.00 (0.95)	−0.08 (0.131)	0.566
Workplace									
Living unit	−0.18 (0.88)	Reference		−0.17 (0.90)	Reference		−0.12 (0.87)	Reference	
Activity centre	0.46 (0.71)	0.65 (0.153)	<0.001*	0.53 (0.79)	0.71 (0.155)	<0.001*	0.49 (0.76)	0.61 (0.159)	<0.001*
Both	0.13 (0.99)	0.32 (0.202)	0.118	0.23 (0.85)	0.41 (0.205)	0.047*	−0.01 (1.34)	0.10 (0.211)	0.628
Training in PA support									
No	−0.17 (0.89)	Reference		−0.06 (0.91)	Reference		−0.09 (0.95)	Reference	
Yes	0.49 (0.81)	0.65 (0.134)	<0.001*	0.29 (0.91)	0.35 (0.141)	0.014*	0.42 (0.81)	0.52 (0.141)	<0.001*
Internal support									
No	−0.18 (0.92)	Reference		−0.13 (0.89)	Reference		−0.04 (0.95)	Reference	
Yes	0.15 (0.89)	0.33 (0.125)	0.010*	0.16 (0.93)	0.29 (0.126)	0.023*	0.10 (0.93)	0.14 (0.130)	0.280
PA plan for people with ID									
No	−0.15 (0.95)	Reference		−0.19 (0.85)	Reference		−0.10 (0.90)	Reference	
Yes	0.36 (0.92)	0.51 (0.152)	<0.001*	0.35 (1.02)	0.54 (0.155)	<0.001*	0.30 (0.91)	0.41 (0.157)	0.011*
Mixed	−0.07 (0.78)	0.08 (0.164)	0.636	0.07 (0.87)	0.26 (0.166)	0.120	0.02 (1.00)	0.13 (0.169)	0.460
Age		0.02 (0.005)	<0.001*		0.02 (0.005)	<0.001*		0.01 (0.005)	0.017*
Duration of contact		−0.00 (0.010)	0.736		−0.01 (0.010)	0.544		−0.01 (0.010)	0.149
Work experience		0.01 (0.006)	0.016*		0.02 (0.006)	0.002*		0.01 (0.006)	0.057*

DSP, direct support professional; ID, intellectual disability; PA, physical activity.

* *P* ≤ 0.10.

Univariate analyses of the capability score revealed significant associations (*P* value ≤ 0.10) with age, work experience, physical activity training, workplace, internal support and the availability of physical activity plans. Small positive effects on the capability scores were found for age and work experience with people with ID. The results also showed that the capability scores were higher on average in participants who received additional training in physical activity support, in participants who were advised by a physical therapist or movement therapist within their organisation (i.e. internal support), in participants with physical activity plans for all the people they supported and in participants working at an activity centre.

Significant associations was also found between opportunity and age, work experience, educational level, physical activity training, workplace, internal support and the availability of physical activity plans. The opportunity score was also lower on average among participants with higher professional education or a master's degree background than in the participants with vocational educational backgrounds.

Univariate analyses of the motivation scores showed significant associations with age, work experience, physical activity training, workplace and the availability of physical activity plans.

### Final models

Table 4 presents the results of the final multivariate linear regression models for the three subscales. Work experience was excluded because of interdependence with the direct support professionals' age and to minimise any multicollinearity problems.

**Table 4:** Final multivariate regression models for capability, opportunity and motivation in direct support professionals

Explanatory variables	Capability		Opportunity		Motivation	
	Multivariate		Multivariate		Multivariate	
	Coefficient (SE)	*P*	Coefficient (SE)	*P*	Coefficient (SE)	*P*
Intercept	−1.25 (0.232)	<0.001	−1.01 (0.245)	<0.001	−0.74 (0.371)	0.047
Age DSP	0.02 (0.005)	<0.001	0.02 (0.005)	0.004	0.01 (0.009)	0.298
Training in PA support (yes)	0.56 (0.140)	<0.001			0.47 (0.150)	0.002
Workplace (activity centre)	0.58 (0.150)	<0.001	0.71 (0.158)	<0.001	0.56 (0.163)	0.001
Workplace (both)	0.19 (0.202)	0.352	0.41 (0.214)	0.058	0.04 (0.217)	0.846
PA plan (yes)	0.37 (0.145)	0.012	0.40 (0.152)	0.008	−0.90 (0.602)	0.135
PA plan (half of the group)	−0.03 (0.153)	0.851	0.11 (0.163)	0.498	0.44 (0.586)	0.454
Age DSP × PA plan (yes)					0.03 (0.014)	0.042
Age DSP × PA plan (half of the group)					−0.01 (0.013)	0.534
Model adjusted *R* ^2^	0.247		0.167		0.178	

DSP, direct support professional; PA, physical activity.

The results show that training in physical activity support was associated with the direct support professionals' capability and motivation. In general, the expected latent trait scores were increased by a 0.56 point for capability and a 0.47 point for motivation for the participants who had received additional training in physical activity support compared with those who did not. The results also demonstrate that the workplace was associated with the direct support professionals' capability, opportunity and motivation. Overall, the expected latent trait scores for capability, opportunity and motivation were increased by 0.58, 0.71 and 0.56 points, respectively, for activity centres compared with living units.

Similarly, having or not having physical activity plans for all the people in care contributes to large differences in the direct support professionals' capability, opportunities and motivation. Participants would be expected to score 0.37 and 0.40 points higher, respectively, for capability and opportunity when physical activity plans are available for all the people with ID their workplace, compared with places lacking these plans. We found an interaction effect in the motivation scores between the direct support professionals' age and whether they had physical activity plans for all the people they support. A small but significant coefficient showed that the positive effect of direct support professionals' age on the motivation scale was predominantly observed where they had physical activity plans for all the people they support (coefficient = 0.03, SE = 0.014). In addition, the direct support professionals' age had a significantly positive effect on the latent trait scores for capability and opportunities. The older a direct support professional was, the more likely he or she was to be able to engage in physical activity support and to experience fewer barriers in the contextual opportunities.

## Discussion

The purpose of this study was to establish the reasons behind the differences in the levels of capability, opportunity and motivation that direct support professionals report regarding the physical activity support they provide to people with ID. The relationship between the components of behaviour and potential explanatory factors related to the characteristics of the people with ID and the professionals' characteristics were investigated. The results showed that the professionals' characteristics were found to be associated with the components generating their behaviour. The capability and motivation levels of direct support professionals who received additional training in physical activity support appeared to be higher. Working in an activity centre relates to higher levels of capability, opportunity and motivation compared with working in a living unit. We also found that the direct support professionals' age and whether they had physical activity plans for the people with ID are related to the variance in capability, opportunity and motivation. Remarkably, the characteristics of the people with ID, such as their level of ID and the presence of additional motor disabilities, did not contribute to the variance in the direct support professionals' behaviour.

Our results are partly aligned with the results of other studies on professional characteristics that associate with supporting people with ID to engage in healthy daily activities, with notable exceptions. Previous studies have shown that the training of professionals had a positive impact on the quality of support, meaning an increase in the level of engagement in activity in people with severe to profound ID (Jones *et al*. 1999; Jones *et al*. 2001; Mansell *et al*. 2008). In our study, receiving training in supporting physical activity in people with ID was only linked to the direct support professionals' capability and motivation to provide physical activity support. Our study also demonstrated that more experienced direct support professionals score higher on capability and opportunity than less experienced direct support professionals. More experienced direct support professionals also scored higher for motivation in our study, but the significant interaction coefficient showed that this effect was only apparent when physical activity plans were available for all the people with ID at their workplace. This is in line with the results of Felce *et al*. (2002), who found evidence that more experienced professionals predicted higher levels of attention and support provided to people with ID. Another factor which contributed to the direct support professionals' behaviour in our study was workplace. This finding is aligned with the study by Qian *et al*. (2015), who noted that location greatly contributed to explaining the differences in the engagement of people with ID in daily activities. We did not find effects for profession and educational level on the direct support professionals' behaviour regarding the physical activity support they provided to people with ID, in contrast to previous studies (Robertson *et al*. 2000; Mansell *et al*. 2008; Qian *et al*. 2015). This might, however, be explained by the fact that in the Netherlands, where our study was conducted, all direct support professionals have at least a vocational qualification. Direct support professionals without any formal professional qualification participated in the study by Mansell *et al*. (2008). Furthermore, unlike the recommendations made in previous studies (Jones *et al*. 1999; Mansell *et al*. 2003; O'Leary *et al*. 2017), we did not find that receiving internal support contributed to the variance in the direct support professionals' behaviour. Nonetheless, the univariate analysis in our study showed an effect for internal support on the direct support professionals' capability and opportunities.

Our results also showed that the characteristics of people with ID are not significantly related to the variance in the direct support professionals' capability, opportunity and motivation. It is notable that the descriptive statistics indicated that participants scored on average higher for capability, opportunity and motivation when they were supporting people with a more severe levels of ID or people with additional impairments (i.e. motor, visual, auditory and/or health). This is not aligned with the findings presented in our qualitative study, in which direct support professionals expressed that these characteristics predominantly acted as barriers (Bossink *et al. under review*). Likewise, a stakeholder comparison of existing physical activity research in people with ID showed that according to direct support professionals, these characteristics mainly limited the physical activity of people with ID (Bossink *et al*. 2017). This difference might be attributed to the *motivation and/or preferences of the people with ID*, which was not included as an explanatory variable in this study's analyses. The influence of *motivation and/or preference of the people with ID* is generally considered as an obstructing factor. It can be argued that professionals who support people with less severe impairments encounter more difficulties with the individuals' motivation or preferences and also confront the ethical dilemma of supporting physical activity without encroaching on self‐determination and individual autonomy. However, their crucial role in the support of people with ID should not be underestimated. For example, a study by Kuijken *et al*. (2016) showed that adults with mild to moderate ID have a good understanding of the importance of regular exercise for living healthily but face difficulties translating this knowledge into behaviour. Kuijken *et al*. suggest that interventions for adults with mild to moderate ID should also focus on their physical and social environment, such as the support provided by professionals. Moreover, a multicomponent intervention targeting both adults with mild to moderate ID and their professional carers can result in an increase in physical activity along with a change in the working routines of the direct support professionals (Bergstrom *et al*., 2013). Additionally, gender is an explanatory variable that needs to be considered in terms of the results. A systematic review on physical activity levels in adults with ID has demonstrated that woman are at higher risk of being physically inactive compared with men (Dairo *et al*. 2016). However, participants in this study were asked to complete the questionnaire keeping the group of people with ID, they worked with, in mind. Consequently, it was not possible to analyse the influence of gender in this study. Results might have been different if participants had taken one specific individual with ID into account, and future research into this issue is needed.

Our findings have important implications for practice and policy. First, retaining experienced direct support professionals appears to be important. Although the direct support professionals' age is a non‐modifiable factor, the physical activity support for people with ID could benefit from mixed teams in terms of the direct support professionals' age and work experience. Moreover, this recommendation can be strengthened by the fact that social influence is acknowledged as an important part of the opportunities available, one of the core components of the ‘COM‐B system’ (Michie *et al*. 2011). Second, direct support professionals appear to benefit from the availability of physical activity plans for all the people they support. They are more likely to have the necessary knowledge and skills and the opportunities to use those resources. Because the direct support professionals' age and the availability of physical activity plans jointly predict motivation, there is, however, a need to find ways to ensure the these plans also target the motivation of younger, generally less experienced direct support professionals. Third, training in physical activity support appears to have a positive effect on the direct support professionals' capability and motivation. The support can therefore generally be enhanced by providing training in physical activity support for all direct support professionals. However, the findings also imply that existing training programmes do not distinguish between the opportunities afforded. Bearing in mind that opportunity can influence motivation, future training programmes are strongly recommended to include conditions in the physical and social environment of direct support professionals.

As this was a cross‐sectional study, future research should focus on the implementation of these findings and their effects on in the physical activity support provided to people with ID. It would in particular be interesting to target future research at the design of an internal training programme for direct support professionals, including strategies for changing the physical and social environment within the organisational context, and to analyse whether this promotes the support of physical activity in people with ID and their actual levels of activity.

## Source of funding

No external funding was received for the research reported in the paper.

## Conflict of Interest

This research did not receive any specific grants from funding agencies in the public, commercial or not‐for‐profit sectors. There was no conflict of interest, and no restrictions were imposed on the publication of results.
